# Clone-directed therapy for proliferative glomerulonephritis with monoclonal immunoglobulin depositions: is it always necessary? ***Two case reports and literature review***

**DOI:** 10.1007/s40620-020-00723-2

**Published:** 2020-03-27

**Authors:** Rob C. M. van Kruijsdijk, Alferso C. Abrahams, Tri Q. Nguyen, Monique C. Minnema, Joannes F. M. Jacobs, Maarten Limper

**Affiliations:** 1 Department of Nephrology and Hypertension, University Medical Center Utrecht, F03.2.22, P.O. Box 85500, 3508 GA Utrecht, The Netherlands; 2 Department of Pathology, University Medical Center Utrecht, Utrecht, The Netherlands; 3 Department of Hematology, University Medical Center Utrecht, Utrecht, The Netherlands; 4 Department of Laboratory Medicine, Laboratory Medical Immunology, Radboud University Medical Center, Nijmegen, The Netherlands; 5 Department of Rheumatology and Clinical Immunology, University Medical Center Utrecht, Utrecht, The Netherlands

**Keywords:** M-protein, Monoclonal gammopathy of renal significance, MGRS, Proliferative glomerulonephritis with monoclonal immunoglobulin deposits

## Abstract

Monoclonal gammopathy of renal significance (MGRS) encompasses a group of disorders in which a monoclonal immunoglobulin (M-protein) secreted by a B-cell or plasma cell clone causes renal disease. Proliferative glomerulonephritis with monoclonal immunoglobulin deposits (PGNMID) is a form of MGRS where M-protein is deposited in the glomerulus. Although evidence is limited, the current consensus is that therapy for PGNMID should be directed against the underlying clone. However, it is conceivable that there is heterogeneity in the renal prognosis of PGNMID and that not all patients have need for clone-directed therapy. Here, we report two cases of PGNMID with IgM-kappa gammopathy. In one case of a 53-year-old woman the glomerulonephritis resolved without clone-directed therapy. In the other case of a 34-year-old woman clone-directed therapy was discontinued due to adverse effects. Although no hematological response was achieved, the PGNMID resolved. In both cases there are no signs of a recurrent glomerulonephritis in over 3 years of follow-up. Here, we review the literature and suggest that some PGNMID patients have a favorable renal prognosis in whom clone-directed therapy can be withheld or postponed. Further research is warranted to yield predictors to identify these patients and to better understand the disease course of PGNMID.

## Introduction

The term monoclonal gammopathy of renal significance (MGRS) has been coined in 2012 to describe a group of renal disorders caused by a monoclonal immunoglobulin (M-protein) secreted by a nonmalignant plasma cell or B-cell clone [1–3]. Proliferative glomerulonephritis with monoclonal immunoglobulin deposits (PGNMID) is a form of MGRS affecting the glomerulus, often leading to chronic or end-stage kidney disease [4, 5]. Although evidence is limited, the current consensus is that therapy for PGNMID, similar as for other forms of MGRS, should be directed at the underlying clone with the goal to achieve deep hematological response and thereby improve the renal prognosis. However, it is conceivable that there is heterogeneity in the renal prognosis of PGNMID and that not all patients need to undergo this type of treatment. Here, we illustrate this by two cases of PGNMID and by summarizing the available literature.

## Case 1

A 53-year-old woman visited our outpatient clinic because of a systemic lupus erythematosus (SLE)/Sjögren syndrome overlap. Her medication included low-dose prednisolone, hydroxychloroquine, and azathioprine. The latter was recently started as steroid-sparing approach. On a routine check-up 2 weeks after starting azathioprine, urinalysis showed 500 white blood cells (WBC), 300 red blood cells (RBCs)/μL, of which > 40% were dysmorphic, and 0.36 g protein/day. She had no clinical signs of a urinary tract infection and the urine culture was negative. Her serum creatinine level had gradually increased from 0.6 to 0.8 mg/dL in the previous 4 months. Repeat urinalysis 3 weeks later showed no WBCs, but the new onset glomerular hematuria persisted. Serum protein electrophoresis (SPE) and immunofixation revealed a non-quantifiable IgM-kappa M-protein. The serum free-light chain (FLC) ratio (kappa/lambda) was slightly elevated (2.04). Serum cryoglobulins were negative.

**Fig. 1:**
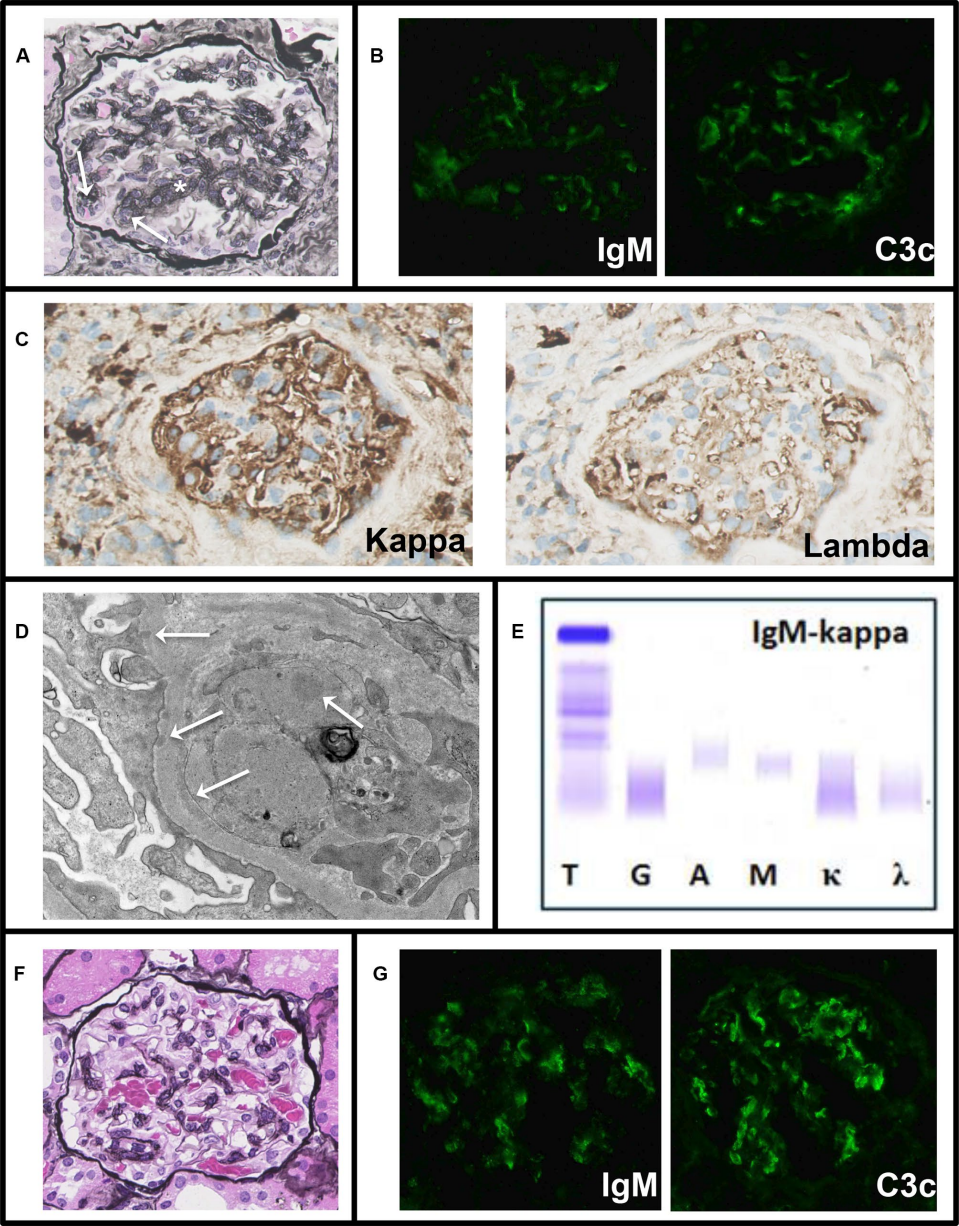
Kidney biopsy and serum immunofixation electrophoresis of patient 1. **a** Biopsy at presentation: Light microscopy showed eight glomeruli of which one was globally and one subtotally sclerosed. The remaining glomeruli showed some mesangial proliferation (asterisk) and five glomeruli showed endocapillary hypercellularity (arrows). The extent of tubular atrophy and interstitial fibrosis was estimated at 10–20%. The arteries and arterioles showed no abnormalities. Congo red staining was negative. **b** Biopsy at presentation: Immunofluorescence showed granular staining in glomeruli for IgM (1+ to 2+) and C3c (2+), while IgG, IgA, kappa- and lambda-free light chains and C1q were negative (not shown). **c** Biopsy at presentation: Immunohistochemistry indicated more intense staining in glomeruli for kappa than for lambda. **d** Biopsy at presentation: Electron microscopy showed subtle deposits in the mesangium, and on the subendothelial and subepithelial side of the glomerular basement membrane (arrows). **e** Serum immunofixation electrophoresis shows IgM-kappa M-protein. **f** Biopsy after 3 years: light microscopy showed no more mesangial or endocapilary hypercellularity. **g** Biopsy after 3 years: Immunofluorescence still showed mesangial deposits of IgM and C3c

A kidney biopsy was indicative of a proliferative glomerulonephritis with monoclonal IgM-kappa deposits (Fig. 1a–d). Furthermore, a revision of a lip biopsy from a year before, which had confirmed the Sjögren syndrome diagnosis, showed 62% IgM positive plasma cells with predominance of kappa over lambda and PCR analysis using the IdentiClone IGH gene clonality assay (InVivoScribe Technologies) showed B-cell monoclonality. Bone marrow biopsy showed no infiltration of monoclonal B-cells or plasma cells, and the IdentiClone assay showed no B-cell clonality. Remarkably, 6 weeks after the kidney biopsy, the M-protein was no longer detectable on repeat measurements. Also, the urine sediment normalized and there was no further increase in the serum creatinine level. Hence, no clone-directed therapy was started. Her treatment regimen including azathioprine/prednisolone remained unchanged. Three years later, immunofixation again showed a non-quantifiable IgM-kappa M-protein. Immunofluorescence on a repeat kidney biopsy still showed mesangial IgM-deposits, but no more mesangial or endocapillary hypercellularity was seen (Fig. 1e–f). Up to the time of writing this report, the serum creatinine level remained stable and there was no hematuria or proteinuria.

## Case 2

**Fig. 2:**
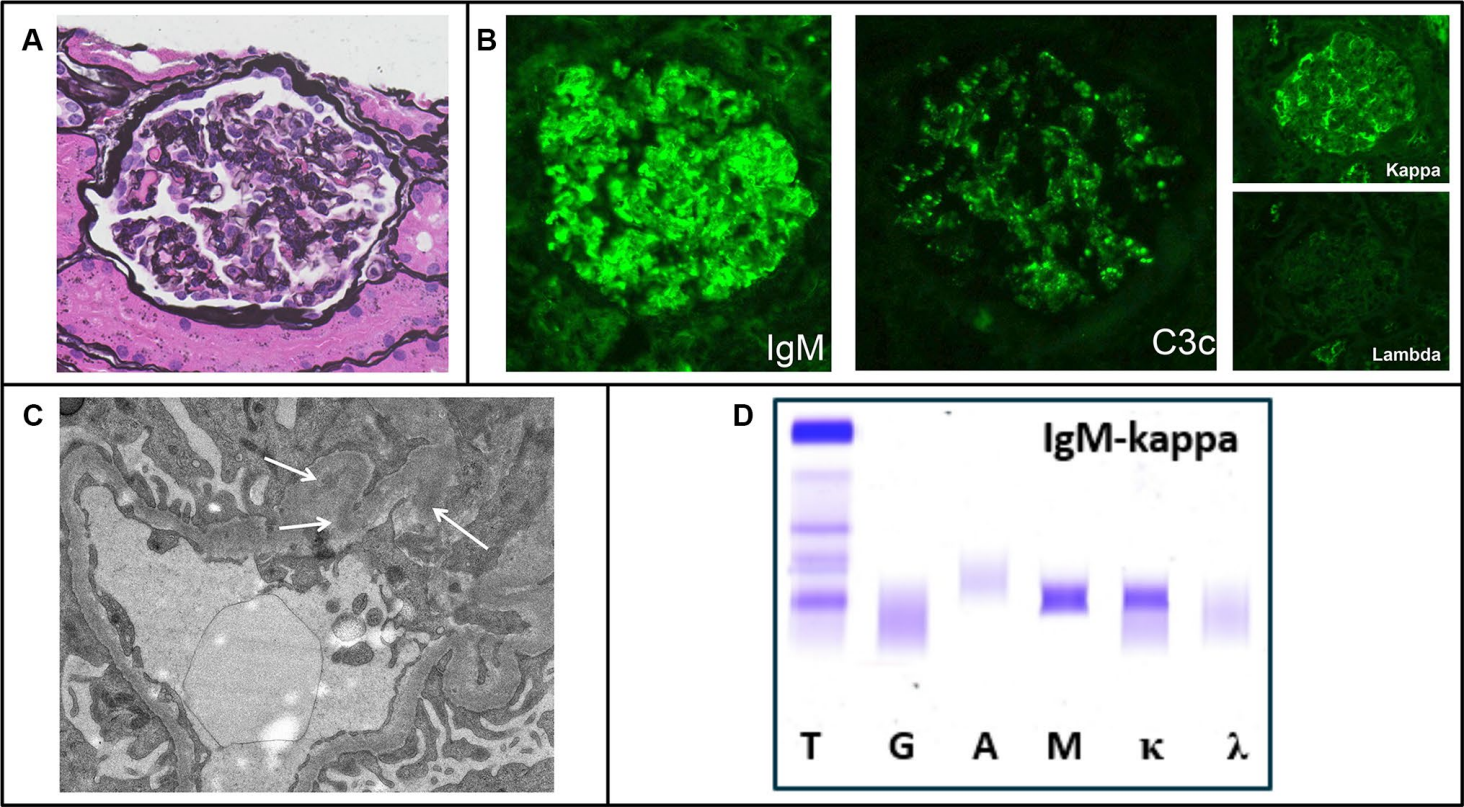
Kidney biopsy and serum immunofixation electrophoresis of patient 2. **a** Light microscopy showed 13 glomeruli of which none was globally sclerosed. All glomeruli showed mild mesangial proliferation without signs of endocapillary hypercellularity. The extent of tubular atrophy and interstitial fibrosis was less than 10%. **b** Immunofluorescence showed intense granular staining in glomeruli for IgM (3+), C3c (1+ to 2+) and kappa (2+), while lambda was negative. **c** Electron microscopy showed subtle deposits in the mesangium (arrows). **d** Serum immunofixation electrophoresis showed IgM-kappa M-protein

A 34-year-old woman presented with microscopic hematuria and proteinuria. Her medical history included a pharyngeal non-Hodgkin lymphoma at the age of 12 and immune thrombocytopenia purpura for which she had a splenectomy. Two years before, an IgM-kappa M-protein of 7 g/L was detected. Although she had thrombocytopenia, low complement levels and positivity for anti phospholipid antibodies, she did not meet the criteria for SLE. At presentation urinalysis showed 150 RBCs/μL (> 40% dysmorphic), RBC casts and 1.02 g protein/day. Serum creatinine was 0.8 mg/dL and the IgM-kappa M-protein level remained stable at 7 g/L. Kidney biopsy showed a proliferative glomerulonephritis with monoclonal IgM-kappa deposits (Fig. 2a–c). About 5% infiltration of IgM-kappa positive lymphoplasmacytic cells was seen in a bone marrow biopsy, the IdentiClone assay showed B-cell clonality. Given the diagnosis of PGNMID treatment with bortezomib, rituximab, and dexamethasone was initiated. The rituximab was discontinued after the first cycle because of adverse events. After receiving five cycles of bortezomib and dexamethasone the treatment was stopped because of progressive polyneuropathy. The M-protein had decreased to 4 g/L. Although no complete hematological response was achieved, the hematuria and proteinuria had resolved and the serum creatinine remained stable. Soon after discontinuation of the treatment the IgM-kappa M-protein progressed to the initial level of 7 g/L (Fig. 2d). As the M-protein level remained stable thereafter a watchful-waiting approach was taken. At the time of writing this report, over 3 years after the kidney biopsy, there are no signs of a recurrent glomerulonephritis.

## Discussion

In PGNMID M-protein is deposited in the glomerulus, mostly causing membranoproliferative lesions, occasionally only with mesangial proliferation, on light microscopy [2, 5]. Immunofluorescence generally shows a granular staining pattern in the glomeruli restricted to a single immunoglobulin heavy chain (mostly IgG) and light chain subtype, and non-organized mesangial and subendothelial deposits can be seen in electron microscopy. While there is limited evidence, there is consensus that treatment of MGRS should target the underlying clone, since complete hematological response is associated with the best renal outcomes [1, 6]. However, in contrast to other forms of MGRS, the detection rate of serum M-protein in PGNMID is only 32–37% and a pathologic clone is found in a bone marrow biopsy in only 25–42% of cases [6–8]. Nonetheless, empirical treatment prescribed to target a hypothesized underlying clone is associated with renal response in cases without a detectable clone [6].

In case 1, the bone marrow biopsy showed no pathologic clone, but possibly the detected M-protein could have been produced by a precursor of a mucosa-associated lymphoid tissue (MALT)-lymphoma, given the abundant IgM-kappa positive plasma cells and the finding of B-cell monoclonality in the lip biopsy. MALT-lymphomas frequently produce M-protein, particularly IgM-kappa [9]. Even though no clone-directed treatment was given, the M-protein and the glomerulonephritis resolved. Previous studies show that in some patients with monoclonal gammopathy of undetermined significance (MGUS) the M-protein disappears without apparent cause [10, 11]. The probability of M-protein persistence seems to depend on the quantity, as in patients with a quantifiable M-protein only 0.4% disappeared spontaneously [10], whereas in patients with an M-protein without quantifiable M-spike 16% disappeared without immunomodulating treatment [11]. The fact that in the present case the M-protein was detectable again after 2 years strongly suggests that the responsible clone did not completely disappear. The question remains whether the M-protein disappeared spontaneously or that the prednisolone or azathioprine attenuated the PGNMID in an early stage. Renal response on immunosuppressive therapy has been described in various cases of MGRS, but small retrospective studies suggest that clone-directed regimens result in higher renal response rates [6, 12, 13].

In the second case, the PGNMID resolved even though no complete or sustained hematological response was achieved. Two similar cases with complete renal response while the M-protein was still detectable after clone-directed therapy were described in a recent case series [6], indicating that renal response is not contingent on the resolution of the M-protein in PGNMID. The fact that the M-protein increased to the pretreatment level without any signs of recurrent glomerulonephritis suggests that a factor besides the M-protein presence or quantity is involved in the development of PGNMID. Possibly, specific characteristics of the M-protein and its interaction with the patient’s immune system play a role. The question remains whether the trigger for PGNMID disappeared due to the clone-directed therapy or that it would have also disappeared without therapy. Either way, it challenges the current assumption that deep hematological response should be pursued with clone-directed therapy in order to achieve the most favorable renal outcome. In fact, other cases of PGNMID with favorable renal outcomes without receiving clone-directed therapy have been described [4, 6, 8]. Among 65 patients from three case series together with our patients (summarized in Table 1), 73% of the patients who received clone-directed therapy had complete or partial renal response, but also 54% of the patients who received steroids, mycophenolate mofetil and/or cyclosporine, and 29% of the patients who received no treatment or renin-angiotensin system-blockade alone achieved complete or partial renal responses [4, 6, 8]. While PGNMID typically presents with overt proteinuria [4, 6, 8], our patients had relatively mild proteinuria, which might be a predictor of favorable renal prognosis. The International Kidney and Monoclonal Gammopathy Research Group recommends careful surveillance in patients with stages 1 and 2 chronic kidney disease (CKD) without evidence of progression and proteinuria < 1 g/day [14]. However, there are also patients with more severe proteinuria who have complete renal response without clone-directed therapy (Table 1). As expected, severe interstitial fibrosis and tubular atrophy is related to worse renal outcome. Among patients with a clone, all who received clone-directed therapy showed renal response, whereas those who received non-directed therapy had no or partial renal response. In contrast, all patients without a detectable clone who were treated with non-directed therapy had partial or complete renal response. Of course, the small sample size and risk for confounding by indication limit the conclusions that can be made from these observational studies.

**Table 1:** Summarized characteristics of the presented cases and 65 cases from previously published case series of PGNMID

Renal response	ESKD (%)	Treatment	*N*	Follow-up in months	eGFR	Proteinuria (g/day)	IFTA^c^				Type of Ig^d^			Serum M-protein detected? (%)	Clone detected?
							None (%)	Mild (%)	Moderate (%)	Severe (%)	IgG (%)	IgM (%)	IgA (%)		
Complete	0	Clone-directed therapy^a^	9	26 (7–96)	52 (23–83)	3.1 (1.9–6.7)	10	70	10	0	89	11	0	80	56%
Complete	0	Non-directed therapy^b^	4	30 (25–36)	34 (7–75)	5.6 (0.4–9.0)	0	75	25	0	75	25	0	25	0%
Complete	0	RAS-blockade alone/none	3	67 (11–114)	75 (40–109)	5.8 (3.8–7.8)^e^	67	33	0	0	100	0	0	33	33%
Partial	0	Clone-directed therapy^a^	15	29 (3–106)	49 (11–127)	3.2 (0.6–15.0)	0	73	20	7	80	13	7	13	13%
Partial	0	Non-directed therapy^b^	3	15 (14–81)	18 (12–62)	4.3 (3.5–5.0)	0	67	0	33	100	0	0	67	33%
Partial	0	RAS-blockade alone/none	3	8 (4–44)	68 (46–127)	3.4 (0.4–3.5)	33	67	0	0	100	0	0	0	NA
None	33	Clone-directed therapy^a^	9	68 (34–101)^e^	60 (20–68)	14.0 (3.9–24)^e^	0	75	0	25	89	11	0	50	0%
None	33	Non-directed therapy^b^	6	NA	39^e^	5.9^e^	0	0	0	100	100	0	0	0	100%
None	47	RAS-blockade alone/none	15	9 (6–10)	36 (14–80)	8.0 (0.4–8.5)	17	17	33	33	93	7	0	17	33%

Summary of case characteristics from Nasr et al. [4] (complete response defined as remission of proteinuria to < 500 mg/day with normal renal function and partial response as reduction in proteinuria by at least 50% and to < 2 g/day with stable renal function), Gumber et al. [6] (complete response defined as stabilization or improvement in eGFR and urine proteinuria improvement to < 0.5 g/g on urine protein-to-creatinine ratio or < 0.5 g/24-h urine collection and partial response as stabilization or improvement of eGFR, but not to normal, and > 50% decrease in proteinuria) and Kousios et al. [8] (complete response defined as stable or improved eGFR and urine protein-to- creatinine ratio < 50 mg/mmol and partial response as eGFR stable or improvement but not back to normal and > 50% reduction in urine protein-to-creatinine ratio)

Data are presented as median (range) or percentages. Percentages may not add up because of rounding

*PGNMID* proliferative glomerulonephritis with monoclonal immunoglobulin deposits, *RAS* renin-angiotensin system, *eGFR* baseline glomerular filtration rate estimated using the Modification of Diet in Renal Disease-4 formula, *uPCR* urine protein-to-creatinine ratio, *IFTA* interstitial fibrosis and tubular atrophy, *Ig* immunoglobulin, *ESKD* end stage kidney disease, *NA* not available

^a^Clone-directed therapy, or empirical therapy if no clone was detected. Regimens included combinations with rituximab, cyclofosfamide, bortezomib, dexamethasone, prednisolone, chlorambucil, mycofenolate mofetil and/or thalidomide

^b^None-directed therapy: prednisolone alone or in combination with mycofenolate mofetil/cyclosporine

^c^IFTA: mild < 25%; Moderate 25–50%; severe > 50%

^d^Type of immunoglobulin heavy chain detected with kidney biopsy immunofluorescence

^e^Data from ≤ 2 cases was available

Both our patients had IgM deposits, whereas the majority of PGNMID patients have IgG deposits, which is a similar distribution as in MGUS [10]. Although the clinicopathologic characteristics, clone and serum M-protein detection rates do not significantly differ between IgG and non-IgG PGNMID [7], it is yet to be determined whether the type of immunoglobulin heavy chain affects the renal prognosis in PGNMID.

It remains a challenge to predict which patients with PGNMID have a favorable renal outcome without the need for clone-directed therapy. Future research into the pathophysiology and predictors of renal prognosis in PGNMID might help to allocate the right treatment to the right patient. Meanwhile, treatment for MGRS should be patient-tailored, preferably by consulting a multidisciplinary team consisting of nephrologists, hematologists and pathologists [3].
